# Factors associated with initiation and completion of the quadrivalent human papillomavirus vaccine series in an ontario cohort of grade 8 girls

**DOI:** 10.1186/1471-2458-11-645

**Published:** 2011-08-13

**Authors:** Leah M Smith, Paul Brassard, Jeffrey C Kwong, Shelley L Deeks, Anne K Ellis, Linda E Lévesque

**Affiliations:** 1 Department of Community Health and Epidemiology, Queen's University, Carruthers Hall, Kingston, ON, K7L 3N6, Canada; 2 Kingston, Frontenac, Lennox, & Addington Public Health, 221 Portsmouth Avenue, Kingston, ON, K7M 1V5, Canada; 3 Division of Clinical Epidemiology, McGill University Health Center, 687 Pine Avenue West, Montreal, QC, H3A 1A1, Canada; 4 Department of Medicine, McGill University, McIntyre Building, 3655 Promenade Sir William Osler, Montreal, QC, H3G 1Y6, Canada; 5 Institute for Clinical Evaluative Sciences, 2075 Bayview Avenue, Toronto, ON, M4N 3M5, Canada; 6 Department of Family and Community Medicine, University of Toronto, 500 University Avenue, Toronto, ON, M5G 1V7, Canada; 7 Dalla Lana School of Public Health, University of Toronto, 155 College Street, Health Science Building, Toronto, ON, M5T 3M7, Canada; 8 Public Health Ontario, 480 University Avenue, Suite 300, Toronto, ON, M5G 1V2, Canada; 9 Division of Allergy and Immunology, Department of Medicine, Queen's University, Doran 1, Kingston General Hospital, 76 Stuart Street, Kingston, ON, K7L 2V7, Canada; 10 Department of Biomedical and Molecular Sciences, Queen's University, Botterell Hall, 20 Stuart Street, Kingston, Ontario, K7L 3N6, Canada

**Keywords:** human papillomavirus vaccine, cohort studies, vaccine acceptability, vaccination, immunization

## Abstract

**Background:**

Although over a hundred million dollars have been invested in offering free quadrivalent human papillomavirus (HPV) vaccination to young girls in Ontario, there continues to be very little information about its usage. In order to successfully guide future HPV vaccine programming, it is important to monitor HPV vaccine use and determine factors associated with use in this population.

**Methods:**

Linking administrative health and immunization databases, we conducted a population-based, retrospective cohort study of girls eligible for Ontario's Grade 8 HPV vaccination program in Kingston, Frontenac, Lennox, and Addington. We determined the proportion of girls who initiated (at least one dose) and completed (all three doses) the vaccination series overall and according to socio-demographics, vaccination history, health services utilization, medical history, and program year. Multivariable logistic regression was used to estimate the strength of association between individual factors and initiation and completion, adjusted for all other factors.

**Results:**

We identified a cohort of 2519 girls, 56.6% of whom received at least one dose of the HPV vaccine. Among vaccinated girls, 85.3% received all three doses. Vaccination history was the strongest predictor of initiation in that girls who received the measles-mumps-rubella, meningococcal C, and hepatitis B vaccines were considerably more likely to also receive the HPV vaccine (odds ratio 4.89; 95% confidence interval 4.04-5.92). Nevertheless, HPV vaccine uptake was more than 20% lower than that of these other vaccines. In addition, while series initiation was not influenced by income, series completion was. In particular, girls of low income were the least likely to receive all three indicated doses of the HPV vaccine (odds ratio 0.45; 95% confidence interval 0.28-0.72).

**Conclusions:**

The current low level of HPV vaccine acceptance in Kingston, Frontenac, Lennox, and Addington will likely have important implications in terms of the health benefits and cost-effectiveness of its publicly funded program. We identified important factors associated with series initiation and completion that should be considered in efforts to improve HPV vaccine use in this population.

## Background

In July 2006, Health Canada approved Gardasil^®^, a quadrivalent human papillomavirus (HPV) vaccine designed to protect against strains of HPV that cause 70% of cervical cancers [1] and 90% of genital warts [2]. The following year, the Canadian government allocated $300 million to the provinces and territories on a per-capita basis to launch a national HPV immunization initiative for young girls [3]. Accordingly, the Ontario government received $117 million and, by September 2007, implemented a program offering free HPV vaccination to all Grade 8 girls in the province. The program is administered by each of Ontario's 36 local public health agencies (LPHAs) primarily through school-based immunization clinics and provides all three indicated doses of the quadrivalent HPV vaccine. Since HPV vaccination is not mandatory, the decision of whether or not to receive the vaccine is left to the parents/guardians of eligible girls [4].

While the initial goal of the Ontario government was to immunize 80% of eligible girls during the program's first two years [5], only 53% received the first dose in year 1 [3]. This level of vaccine coverage is not only considerably lower than that of similar school-based vaccination programs in the province [6], but also represents the lowest known level of HPV vaccine acceptance in Canada [7]. The reasons for such low coverage in Ontario remain unknown; however, surveys have consistently reported that parental concerns about the safety and long-terms effects of the HPV vaccine affect intent to vaccinate [8-12]. As a result, clinical factors, such as medical history and health services utilization, may be important predictors of HPV vaccine receipt. For instance, parents of a girl with a serious health condition may be more reluctant to consent to having their daughter receive a new vaccine for fear that it might further impact their child's health. On the other hand, increased contact with healthcare providers (e.g., physicians) may lead to increased opportunities to discuss the benefits of vaccination, thereby increasing HPV vaccine acceptance and use.

Given that low vaccine coverage undermines public health efforts aimed at reducing the burden of HPV-related illnesses and threatens the program's cost-effectiveness [13,14], it is important to monitor use of the HPV vaccine and identify factors that influence use in order to inform future HPV immunization programs. To this end, we conducted a population-based, retrospective cohort study of girls residing in Kingston, Frontenac, Lennox, and Addington (KFL&A) who were eligible for Ontario's Grade 8 HPV vaccination program in 2007-08 and 2008-09 to describe HPV vaccine use and to identify socio-demographic and clinical factors associated with this use.

## Methods

This study was approved by the Research Ethics Boards of Queen's University and Sunnybrook Health Sciences Centre.

### The KFL&A Grade 8 HPV vaccination program

KFL&A Public Health offers the three indicated doses of the quadrivalent HPV vaccine free-of-charge to all Grade 8 girls in its jurisdiction. These doses are typically administered by public health nurses in school-based immunization clinics held in September/October, November/December, and March/April of each year to correspond with the recommended 0-, 2-, and 6-month dosing schedule of the vaccine. Eligible girls also have the option of obtaining the vaccine for free at their LPHA or through their family physician at their convenience. Under the publicly funded program, girls have until the end of August of their Grade 8 year to initiate the dosing schedule and until the end of their Grade 9 year to complete it.

### Data sources

The study population and factors of interest were identified using Ontario's administrative health databases and the KFL&A Immunization Record Information System (IRIS). The health databases used were: (1) the Registered Persons Database (RPDB) for information on dates of health insurance coverage and socio-demographics, (2) the Canadian Institute for Health Information's (CIHI) Discharge Abstract Database (DAD) for dates of hospitalizations and discharge diagnoses (coded using the International Classification of Diseases, Ninth and Tenth Revisions [ICD-9 and ICD-10]), (3) the National Ambulatory Care Reporting System (NACRS) database for dates of emergency department visits and corresponding diagnoses (coded using ICD-9 and ICD-10), and (4) the Ontario Health Insurance Plan (OHIP) database for dates and diagnoses of all fee-for-service claims by healthcare providers who claim under the publicly funded system (coded using OHIP diagnostic codes) (Additional File 1: Appendix 1). These databases are generated by the province's universal health insurance programs and are accessible through the Institute for Clinical Evaluative Sciences (ICES). Described elsewhere in detail [15-20], these databases have been used extensively in health research, including in the post-marketing evaluation of drug and vaccine safety [21-23]. In each database, residents of Ontario are represented by a unique encrypted identifier that enables complete record linkage at the level of the individual across databases and time.

The KFL&A IRIS database was used for information on vaccinations. IRIS databases, maintained by each of Ontario's LPHAs, were originally developed by the Ministry of Health and Long-Term Care (MOHLTC) to track and record immunizations mandated under the *Immunization of School Pupils Act (1982) *for all school-aged children in the province [24,25]. In addition to mandatory vaccines, IRIS is used to maintain records on other childhood vaccines, especially those that are publicly funded. As such, IRIS captures individual-level information on all HPV vaccines administered through Ontario's publicly funded program. This information is recorded into IRIS by the LPHA, regardless of whether the vaccine is administered at a school clinic, an LPHA, or a physician's office. Since parents/guardians are required to provide their child's immunization records to the LPHA when the child transfers from a school in a different health region, IRIS records are considered up-to-date for students who move into the area. While this is the first study to use IRIS for research purposes, a recent re-abstraction study demonstrated that the KFL&A IRIS database captured individual girls' HPV vaccination status with high sensitivity (99.8%; 95% CI 99.3-99.9) and specificity (97.7%; 95% CI 96.3-98.7) [26]. On April 23, 2010, a copy of the KFL&A IRIS database was electronically transferred to ICES by means of a secure, monitored, high encryption portal. Record linkage with Ontario's administrative health databases was successful for 95.6% of individuals in the database [27].

### Study design

We conducted a population-based, retrospective cohort study of girls eligible for Ontario's Grade 8 HPV vaccination program in KFL&A. This region was studied because researchers were based at the KFL&A health unit and had authorized access to the KFL&A IRIS database from the KFL&A Medical Officer of Health. The RPDB and KFL&A IRIS databases were used to identify girls eligible for the 2007-08 and 2008-09 HPV immunization program years. Since grade at which an individual is immunized is not recorded in IRIS, we defined the study cohort based on birth year and assumed all girls were in Grade 8 thirteen years after their birth year. Accordingly, we identified a cohort of girls born in 1994 and 1995 who were residing in KFL&A on September 1 of 2007 and 2008, respectively. Girls were followed from September 1 of their Grade 8 school year (*cohort entry*) until the earliest of: date of death or April 13, 2010 (*end of study*).

### HPV vaccination history

We ascertained data on HPV vaccinations any time up to and including the study end date. Cohort members who initiated the vaccine series were classified as *vaccinated *(at least one dose) and those who did not were classified as *unvaccinated *(no doses). Vaccinated girls were further classified as *completers *if they received all three doses or *non-completers *if they did not (only one or two doses received). For both birth cohorts, data were available to assess series completion during cohort members' Grade 8 year.

### Baseline characteristics

The socio-demographic factors available for study were age, income, and place of residence. Income and place of residence were ascertained by linking a girl's postal code at cohort entry with a measure of neighbourhood income and community size, respectively, obtained from the 2006 Canadian Census. Neighbourhood incomes were categorized into provincial quintiles and place of residence into urban (≥10,000 persons) or rural (< 10,000) [16].

The clinical profile of each girl consisted of her vaccination history, health services utilization, and medical history.

Data from the KFL&A IRIS database were used to determine vaccination history; that is, if a cohort member had been vaccinated with the measles, mumps, and rubella (MMR; at least two doses of the vaccine), meningococcal C (at least one dose), or hepatitis B (at least two doses) vaccine. We used vaccination history to assess a parent's tendency to accept publicly funded immunization programs on behalf of their child (i.e., a proxy for general vaccine acceptance). *Post priori*, we also considered uptake of all three vaccines (yes/no) as a proxy for consistent pro-vaccine sentiments.

Health services utilization was classified in terms of the number of hospital admissions, emergency department (ED) visits, or outpatient physician visits occurring any time prior to cohort entry (i.e., between birth and cohort entry). Each type of service was considered separately and used as an indicator of health and level of contact with the healthcare system.

We established medical histories by identifying conditions leading to a hospital admission, an ED visit, or a physician visit any time prior to cohort entry. Medical conditions of interest included history of an autoimmune disease, presence of a risk factor for autoimmune diseases, history of an immune-mediated disease, and selected specific illnesses (Additional File 1: Appendix 2). In addition, a composite indicator of other serious illness was identified. The list of medical diagnoses evaluated was developed *a priori *in consultation with a clinical immunologist and two Public Health physicians. This list included conditions that could predispose a girl to an adverse event following HPV immunization (e.g., immune-mediated conditions) or that, because of their seriousness, could affect the decision to vaccinate against HPV (e.g., epilepsy, cancer).

### Statistical analysis

To describe the patterns of use of the HPV vaccine, we calculated the percentage of vaccinated and unvaccinated girls (series initiation) and the percentage of completers and non-completers (series completion) in the KFL&A cohort. Subsequently, these analyses were stratified by program year. We used cross-tabulations to determine the distribution of the various socio-demographic and clinical characteristics according to series initiation and completion and multivariable logistic regression to estimate the strength of these associations, adjusted for all other baseline characteristics.

All statistical analyses were conducted using SAS 9.2 (SAS Institute Inc., Cary, NC).

## Results

**Figure 1:**
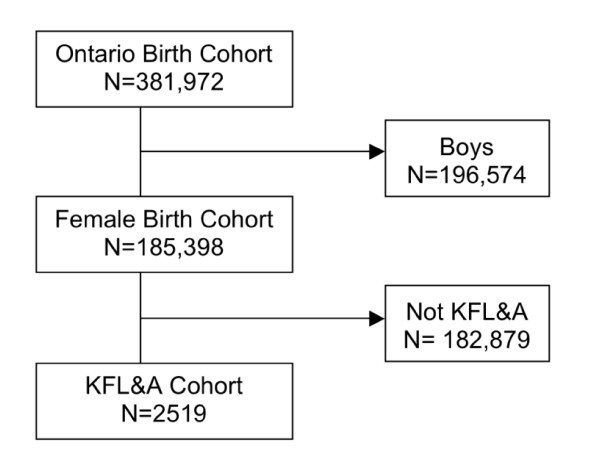
**Cohort flow diagram**.

Based on birth year, we determined that 185,398 girls were eligible for Ontario's Grade 8 HPV vaccination program in its first two years (Figure 1). Of these, 2519 (1.36%) were residing in KFL&A and comprised our study cohort. Cohort members were between 12.7 and 13.6 years of age at cohort entry.

**Figure 2:**
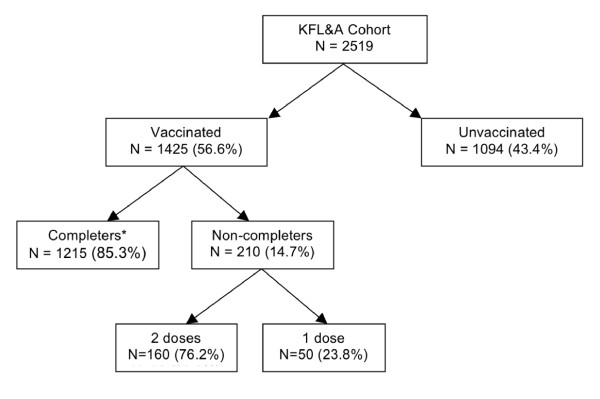
**Patterns of use of the quadrivalent HPV vaccine**. * This group includes two girls who received four doses.

Overall, 56.6% (n = 1425) of eligible girls received at least one dose of the HPV vaccine and less than half (48.2%) received all three (Figure 2). Among vaccinated girls, 85.3% (n = 1215) completed the three dose series, while 11.2% (n = 160) received two doses and 3.5% (n = 50) received only the first.

Series initiation increased significantly across campaign years (OR = 1.29; 95% CI 1.08-1.53), but the increase corresponded to an absolute difference of only 4.0% (Table 1). While there was no significant change in series completion between program years (OR = 0.88; 95% CI 0.65-1.20), the proportion of non-completers receiving two doses was higher in the second year of the program compared to the first (13.1% *vs*. 9.4%; *p *= 0.03).

**Table 1:** Patterns of use of the quadrivalent HPV vaccine according to program year

HPV vaccination status	2007/08 N (%)	2008/09 N (%)	Adjusted OR* (95% CI)	P-value
**Series Initiation**				
Vaccinated	721 (54.7)	704 (58.7)	1.29 (1.08-1.53)	0.04^†^
Unvaccinated	598 (45.3)	496 (41.3)		
**Series Completion**				
Completers^††^	623 (86.4)	592 (84.1)	0.88 (0.65-1.20)	0.22
Non-Completers	98 (13.6)	112 (15.9)		
Received 1 dose	30 (4.2)	20 (2.8)	N/A	0.03^†^
Received 2 doses	68 (9.4)	92 (13.1)		

OR = Odds Ratio; CI = Confidence Interval; N/A = Not Available

* Adjusted for socio-demographics, vaccination history, health services utilization, and medical history

(See Table 2 for details).

† Statistically significant (p < 0.05)

†† Includes two girls who received four doses of the vaccine.

After adjustment, there were no statistically significant differences between vaccinated and unvaccinated girls with respect to health services utilization, medical history, or socio-demographics, except girls in the fourth income quintile were less likely to receive the HPV vaccine than those in the third quintile (Table 2). Conversely, compared to their unvaccinated counterparts, HPV vaccinated girls were significantly more likely to have received the MMR (OR = 3.21; 95% CI 2.25-4.57), meningococcal C (OR = 2.16; 95% CI 1.64-2.84), or hepatitis B (OR = 2.86; 95% CI 2.14-3.83) vaccine and were even more likely to have received all three vaccines (OR = 4.89; 95% CI 4.04-5.92).

**Table 2:** Baseline characteristics of girls vaccinated and unvaccinated with the quadrivalent HPV vaccine

Characteristic *	Vaccinated (N = 1425)	Unvaccinated (N = 1094)	**Adjusted OR **^†^ (95% CI)
**Socio-demographics**			
Age (mean ± SD)	13.2 ± 0.3	13.2 ± 0.3	1.10 (0.81-1.50)
Neighbourhood income quintile			
1 (lowest)	18.9	20.8	0.87 (0.65-1.15)
2	17.5	18.9	0.80 (0.60-1.06)
3 (reference)	20.8	18.1	1.00 (reference)
4	20.4	21.3	0.75 (0.57-0.99)^††^
5 (highest)	21.8	19.4	0.91 (0.69-1.19)
Missing^‡^	0.6	1.5	1.34 (0.50-3.61)
Place of residence			
Urban (reference)	78.4	77.5	1.00 (reference)
Rural	21.1	21.0	1.14 (0.90-1.43)
Missing^‡^	0.6	1.5	1.34 (0.50-3.61)
**Vaccination History**			
MMR	96.1	78.9	3.21 (2.25-4.57)^††^
Meningococcal C	88.5	60.2	2.16 (1.64-2.84)^††^
Hepatitis B	90.7	62.2	2.86 (2.14-3.83)^††^
All three vaccines^§^	83.2	51.1	4.89 (4.04-5.92)^††^
**Health Services Utilisation**			
Hospitalizations^‖^			
None (reference)	19.5	20.4	1.00 (reference)
1	61.9	59.7	0.82 (0.63-1.07)
2	13.1	13.6	0.71 (0.50-1.01)
≥ 3	5.5	6.3	0.64 (0.38-1.06)
Emergency Department visits^‖^			
None (reference)	25.9	30.2	1.00 (reference)
1	21.5	22.2	1.00 (0.78-1.28)
2-7	43.4	38.9	1.16 (0.92-1.46)
≥ 8	9.2	8.7	1.13 (0.77-1.66)
Outpatient visits^‖^			
0-13 (reference)	8.5	12.0	1.00 (reference)
14-45	40.8	42.3	0.91 (0.64-1.31)
46-92	39.9	37.5	1.07 (0.73-1.57)
≥ 93	10.8	8.2	1.31 (0.82-2.11)
**Medical History**			
Previous diagnosis			
Infectious and parasitic diseases	15.2	13.9	0.96 (0.70-1.32)
Intestinal infections	3.7	3.3	1.25 (0.71-2.20)
Chickenpox	1.6	1.7	0.92 (0.44-1.91)
Cancer	0.9	0.6	1.55 (0.52-4.60)
Metabolic disorders	3.0	3.0	0.90 (0.50-1.61)
Disorders of the immune system	0.1	0.1	2.18 (0.07-73.24)
Diseases of the blood	1.4	1.6	0.91 (0.42-1.98)
Mental disorders	0.5	0.7	1.24 (0.38-4.03)
Epilepsy, seizures, and convulsions	3.2	3.1	1.11 (0.67-1.86)
Gastroenteritis	5.5	4.3	1.10 (0.71-1.72)
Nephritis	3.9	3.4	1.24 (0.75-2.04)
Congenital anomalies	7.0	8.9	0.78 (0.56-1.09)
Syncope	1.2	1.3	1.05 (0.47-2.35)
Other serious illness	2.0	1.8	1.05 (0.55-2.00)
Immune-mediated events			
Allergic rhinitis	0.2	0.4	0.53 (0.10-2.87)
Asthma	5.6	6.0	0.96 (0.64-1.44)
Dermatitis	4.3	3.0	1.46 (0.89-2.40)
Autoimmune diseases			
Diabetes mellitus	1.3	1.7	0.57 (0.28-1.15)
Multiple sclerosis	0.0	0.1	-
Bell's palsy	0.2	0.5	0.38 (0.08-1.69)
Guillain-Barré Syndrome	0.0	0.0	-
Juvenile Arthritis	2.2	1.7	1.28 (0.66-2.44)
Systemic Lupus Erythematosus	0.0	0.0	-
Risk factors for autoimmune diseases			
Cytomegalovirus^¶^	0.0	0.1	-
Epstein-Barr virus^¶^	0.2	0.1	4.29 (0.26-71.21)
Campylobacter	0.0	0.0	-
Influenza	15.5	13.2	1.13 (0.88-1.46)
Mycoplasma pneumoniae	0.1	0.1	2.81 (0.09-87.47)

OR = Odds Ratio; CI = Confidence Interval; SD = Standard deviation; MMR = Measles, mumps and rubella

* Ascertained anytime preceding cohort entry; expressed as percentages (%) unless otherwise indicated.

† Adjusted for all characteristics listed in table as well as vaccination program year.

^†† ^Statistically significant (p < 0.05).

‡ Missing because postal code in RPDB was either incorrect or unavailable

§ This variable was defined post-hoc and is adjusted for all factors listed except prior use of the MMR, meningococcal C, and hepatitis B vaccines.

‖ Cut points were based on the frequency distribution of the data for each variable.

Similar to series initiation, series completion was not significantly associated with age, health services utilization, or medical history (Table 3). On the other hand, series completion was associated with certain socio-demographic characteristics. For one, girls living in the lowest income neighbourhoods were significantly less likely to complete the three-dose regimen than girls living in middle-income neighbourhoods (OR = 0.45; 95% CI 0.28-0.72). In addition, girls residing in rural areas were significantly more likely to complete their HPV vaccine series than those residing in urban areas (OR = 1.76; 95% CI 1.14-2.74). Although previous MMR, meningococcal C, or hepatitis B vaccination was not significantly associated with HPV vaccine completion, receipt of all three vaccines was (OR = 1.85; 95% CI 1.29-2.65).

**Table 3:** Baseline characteristics of completers and non-completers of the 3-dose regimen of the quadrivalent HPV vaccine

Characteristic *	Completers (N = 1215)	Non-Completers (N = 210)	**Adjusted OR **^†^ (95% CI)
**Socio-demographics**			
Age (mean ± SD)	13.2 ± 0.3	13.2 ± 0.3	1.02 (0.60-1.75)
Neighbourhood income quintile			
1 (lowest)	16.9	31.0	0.45 (0.28-0.72)^††^
2	17.5	17.6	0.80 (0.48-1.33)
3 (reference)	21.5	17.1	1.00 (reference)
4	21.0	16.7	0.98 (0.59-1.62)
5 (highest)	22.7	16.2	1.15 (0.68-1.92)
Missing^‡^	0.4	1.4	0.56 (0.12-2.66)
Place of residence			
Urban (reference)	77.7	82.4	1.00
Rural	21.9	16.2	1.76 (1.14-2.74)^††^
Missing^‡^	0.4	1.4	0.56 (0.12-2.66)
**Vaccination History**			
MMR	96.8	92.4	1.61 (0.81-3.19)
Meningococcal C	90.0	79.5	1.39 (0.83-2.35)
Hepatitis B	92.3	81.9	1.67 (0.95-2.92)
All three vaccines^§^	85.1	72.4	1.85 (1.29-2.65)^††^
**Health Services Utilisation**			
Hospitalizations^‖^			
None (reference)	18.8	23.8	1.00
1	63.1	54.8	1.23 (0.80-1.91)
2	12.9	13.8	1.08 (0.59-1.98)
≥ 3	5.2	7.6	1.04 (0.45-2.37)
Emergency Department visits^‖^			
None (reference)	26.3	23.3	1.00
1	21.5	21.9	0.85 (0.53-1.35)
2-7	43.3	43.8	0.88 (0.58-1.35)
≥ 8	8.9	11.0	0.73 (0.38-1.40)
Outpatient visits^‖^			
0-13 (reference)	7.6	13.8	1.00
14-45	41.2	38.1	1.42 (0.79-2.54)
46-92	40.7	35.7	1.55 (0.83-2.89)
≥ 93	10.5	12.4	1.43 (0.67-3.05)
**Medical History**			
Previous diagnosis			
Infectious and parasitic diseases	14.5	19.1	0.83 (0.48-1.42)
Intestinal infections	3.2	6.7	0.64 (0.28-1.45)
Chickenpox	1.5	2.4	0.80 (0.25-2.59)
Cancer	0.9	1.0	0.80 (0.16-3.98)
Metabolic disorders	3.0	3.3	1.33 (0.50-3.56)
Disorders of the immune system	0.1	0.0	-
Diseases of the blood	1.6	0.5	4.60 (0.56-37.84)
Mental disorders	0.5	0.5	0.84 (0.09-8.36)
Epilepsy, seizures, and convulsions	3.1	3.8	0.89 (0.38-2.07)
Gastroenteritis	5.3	7.1	0.70 (0.36-1.37)
Nephritis	3.5	5.7	0.62 (0.29-1.30)
Congenital anomalies	7.0	7.1	0.96 (0.52-1.78)
Syncope	1.2	1.0	1.62 (0.34-7.77)
Other serious illness	2.1	1.4	1.66 (0.45-6.14)
Immune-mediated events			
Allergic rhinitis	0.3	0.0	-
Asthma	5.4	6.7	0.89 (0.46-1.75)
Dermatitis	4.4	3.3	1.72 (0.72-4.14)
Autoimmune diseases			
Diabetes mellitus	1.3	1.4	0.77 (0.21-2.83)
Multiple sclerosis	0.0	0.0	-
Bell's palsy	0.3	0.0	-
Guillain-Barré Syndrome	0.0	0.0	-
Juvenile Arthritis	1.9	3.8	0.58 (0.24-1.41)
Systemic Lupus Erythematosus	0.0	0.0	-
Risk factors for autoimmune diseases			
Cytomegalovirus^¶^	0.0	0.0	-
Epstein-Barr virus^¶^	0.3	0.0	-
Campylobacter	0.0	0.0	-
Influenza	15.9	13.3	1.26 (0.80-1.97)
Mycoplasma pneumoniae	0.1	0.0	-

OR = Odds Ratio; CI = Confidence Interval; SD = Standard deviation; MMR = Measles, mumps and rubella

* Ascertained anytime preceding cohort entry; expressed as percentages (%) unless otherwise indicated.

† Adjusted for all characteristics listed in table as well as vaccination program year.

†† Statistically significant (p < 0.05).

‡ Missing because postal code in RPDB was either incorrect or unavailable.

§ This variable was defined post-hoc and is adjusted for all factors listed except prior use of the MMR, meningococcal C, and hepatitis B vaccines.

‖ Cut points were based on the frequency distribution of the data for each variable.

## Discussion

Among eligible girls residing in KFL&A, HPV vaccine initiation was low, but completion of the three-dose regimen was high. While initiation increased significantly between the first and second year of the immunization program, the absolute increase was small and vaccine coverage remained far below that required to maximize the health benefits and cost-effectiveness of the publicly funded program. The factor most strongly associated with series initiation was a history of vaccine use. Income was associated with series completion, but not initiation.

Cost has been shown to be an important barrier to HPV vaccine use [28,29]. Therefore, our finding that series initiation was not influenced by income demonstrates the benefit of providing the HPV vaccine through a publicly funded program. At the same time, we found that those in the lowest income quintile were the least likely to receive all three indicated doses of the vaccine, suggesting that inequities in access may exist despite the availability of free immunization. This is particularly important given that low income has also been associated with lower rates of cervical cancer screening [30,31] and higher rates of cervical cancer incidence and mortality [32,33]. Since non-completers may not acquire the full benefits of HPV immunization and girls of low income have increased need for protection against HPV-related infections, low series completion in this population could have important public health implications and requires further investigation.

Although series completion was lower among those of low income, overall, completion was high among vaccinated girls in our study population. This likely reflects the convenience of a school-based program, where a girl who received consent need only be present to receive her scheduled dose of the vaccine. Among non-completers, girls were three times more likely to receive two doses of the HPV vaccine as opposed to only one, suggesting that lack of completion may be due to factors additional to the withdrawal of consent. One reason for the observed pattern may be attributed to the fact that if a girl is absent on the day of a school immunization clinic she must go to her LPHA or family physician to obtain her missed dose. Low income has been consistently related to school absenteeism [34-36] and it is possible that girls of lower income have fewer resources to follow-up on a missed dose at a location other than their school. This hypothesis is consistent with our findings that income is associated with series completion but not initiation.

As expected, a history of vaccine use, measured using both mandatory (MMR) and optional (meningococcal C and hepatitis B) vaccines, was the strongest predictor of HPV vaccine uptake. These findings are consistent with survey data that indicate that parental attitudes and beliefs about vaccines in general predict their attitudes toward the HPV vaccine [8,37]. Nevertheless, in our study cohort, overall initiation of the HPV vaccine (56%) was still well below that of the meningococcal C (76%) and hepatitis B (78%) vaccines that were offered to the same girls just one year before (i.e., in Grade 7). The low uptake of the HPV vaccine compared to these other optional vaccines suggests that factors additional to "anti-vaccine attitudes" are contributing to poor acceptance of the HPV vaccine.

Low use of the HPV vaccine in Ontario has been partially attributed to the program's rapid implementation and to the ban on the promotion of provincially funded programs that was in place at the time of the program's launch because of Ontario's 2007 provincial election campaign [38]. However, since series initiation barely increased in the second year of the program, our results do not support this hypothesis. Although a number of other factors have also been proposed to explain HPV vaccine avoidance (e.g., fear of its negative influence on sexual behaviour, low perceived risk of HPV infection, lack of knowledge about HPV), surveys consistently report parental concerns about the safety and long-term effects of vaccines as important barriers to vaccination [8-12,39].

We were unable to directly assess whether safety concerns affected receipt of the HPV vaccine in our population, but we were able to explore the relationship between medical history and health services utilization and vaccine usage. Although these associations were not statistically significant, there were some interesting trends. First, it appeared that non-initiation was associated with an increasing number of hospitalizations. Since admission to a hospital is a medical rather than personal decision, a high number of hospitalizations likely indicates the presence of a serious health problem. Therefore, as hypothesized, our results suggest that parents of sicker girls may be less likely to consent to HPV vaccination than those of relatively healthy girls. Furthermore, in our study, a high number of outpatient visits was associated with an increased likelihood of receiving the HPV vaccine, suggesting that increased contact with healthcare providers may be related to increased vaccine acceptance. It is important to note that, given the general good health of the age group studied and the corresponding low event rates, we had limited power to assess the influence of these clinical factors on HPV vaccine receipt. As such, these potential associations should be re-examined in a larger sample.

Although a couple of studies have utilized administrative rather than survey data to study the correlates of HPV vaccine use in the United States [40,41], to our knowledge, ours is the first to link a population-based immunization database with administrative health data in the context of a publicly funded program. This provided us with a unique opportunity to report on patterns of use of the HPV vaccine and examine the clinical factors associated with its acceptance in a situation in which cost of vaccination is not an issue. While this study also benefits from the use of validated HPV vaccination data [26], it has a number of important limitations. For one, because school grade was not available in the databases, we used birth year to identify eligible girls. While children are typically in Grade 8 thirteen years after they are born, this is not the case for those who are advanced or held back a school grade. Therefore, we may have included some ineligible girls in our cohort (e.g., girls born in 1995 who were held back a grade) and excluded some eligible girls (e.g., girls born in 1996 who were advanced a grade). Although these errors may have caused us to underestimate the number of girls vaccinated, given the small proportion of girls who are advanced or held back a grade, the proportion affected by this is expected to be small. In addition, although data were available to assess series completion during the Grade 8 school year, we did not have vaccination data after December 31, 2009. As such, series completion for any girl in the 1995 birth cohort who completed her regimen after December 31, 2009 of her Grade 9 year would have been misclassified. Another limitation is that, since the provincial health databases contain only information on services claimed under OHIP, the clinical history of girls who have not resided in Ontario throughout their lifetime or who have received privately funded care may be incomplete. Also, the accuracy of many of the diagnostic codes used to identify medical history has not yet been assessed, nor has the validity of using neighbourhood income as a proxy for household income in this population. Another disadvantage of these administrative health data is that we were unable to consider factors that have previously been identified as possible predictors of HPV vaccine acceptance, such as race/ethnicity, perceived vulnerability to HPV infection, and concerns about the vaccine's long-term effects and impact on sexual behavior [8-11,37]. Since these factors may have introduced residual confounding into our study, the influence of these factors on series initiation and completion now needs to be assessed taking clinical predictors into account. Finally, since KFL&A is a medium-sized health district of Ontario (N = 184,407, 1.5% of the province) with a slightly lower than average median family income ($66,466 *vs*. $69,156) and a low proportion of visible minorities (5.0% *vs*. 23.0%) [42], the generalizability of our results to other regions remains unclear.

## Conclusions

The current low level of HPV vaccine use in KFL&A will likely have important implications in terms of the health benefits and cost-effectiveness of its publicly funded vaccination program. We identified important factors that were associated with series initiation and completion that should be considered in efforts to guide future HPV vaccine programming. For example, girls in the lowest income quintile were the least likely to complete the recommended three-dose regimen, suggesting that program delivery should be modified to improve series completion in vulnerable populations. Furthermore, since vaccine acceptance is known to be related to concerns about the safety and long-term effects of vaccines, our findings of possible associations between HPV vaccine coverage and medical history and health services utilization require further study.

## Competing interests

The authors declare that they have no competing interests.

## Authors' contributions

All authors made substantial contributions to the conception and design of the study. LMS and LEL acquired the data, LMS, LEL, and PB analyzed and interpreted the data, and LMS drafted the manuscript. All authors provided critical revisions of drafts and read and approved the final manuscript.

## Pre-publication history

The pre-publication history for this paper can be accessed here:

http://www.biomedcentral.com/1471-2458/11/645/prepub

## Supplementary Material

Additional file 1 **Appendix 1 and Appendix 2**. Description, use, and time windows for data sources and Diagnoses and corresponding diagnostic codes for baseline medical history. Click here for file
