# Supplementary material for: Factors associated with initiation and completion of the quadrivalent human papillomavirus vaccine series in an ontario cohort of grade 8 girls
Source: BMC Public Health. 2011 Aug 13;11:645. doi: 10.1186/1471-2458-11-645 (PMC3224094; doi:10.1186/1471-2458-11-645)
Supplement: Additional file 1 — Appendix 1 and Appendix 2. Description, use, and time windows for data sources and Diagnoses and corresponding diagnostic codes for baseline medical history. [file 1471-2458-11-645-S1.DOC]

**Appendix 1 Description, use, and time windows for data sources**

| **Database** | **Description** | **Original Data Source** | **Main data elements** | **Information Obtained** | **Use of data/time-windows** | **Diagnostic record** |
| --- | --- | --- | --- | --- | --- | --- |
| **RPDB**  Registered Persons’ Database | Information about anyone who has ever received an Ontario health card number | MOHLTC (enriched by in-house ICES datasets) | - Demographics (e.g., date of birth, sex) - Geographic information (e.g., postal code) - Date of last contact with health care system | - ICES Key Number (IKN; unique identifier anonymously linkable to other individual-level data holdings) - Birth month and year, sex, income quintile, place of residence | - Sex and date of birth for the identification of cohort members through linkage with IRIS - Other demographic and geographic information - January 1994-December 2009 | - N/A |
| **DAD (CIHI)**  Discharge Abstract Database | Record on inpatient hospital activity | CIHI | - Patient demographics (e.g., sex, age) - clinical data (e.g. diagnoses, procedures) - administrative data (e.g. hospital, length of stay) | - IKN - Admission date - Length of stay - Diagnosis code, diagnosis type | - Dates and diagnoses related to hospitalizations for identification of study outcomes - January 1994-March 2009 | - 1-25 diagnoses per admission - 3-4 character ICD-9 codes - 3-4 character ICD-10 codes - 1-character ‘type of diagnosis’ codes |
| **NACRS**  National Ambulatory Care Reporting System | Record on patient visits to hospital emergency departments, same day surgery, and selected outpatient services | CIHI | - patient demographics - clinical data - administrative data - financial data - service-specific data elements for day surgery and emergency | - IKN - Arrival date - Diagnosis code, diagnosis type | - Date and diagnoses related to emergency consultations for identification of study outcomes - July 2000-March 2009 | - 1-10 diagnoses per consultation - 3-4 character ICD-9 codes - 3-4 character ICD-10 codes - 1-character ‘type of diagnosis’ codes |
| **OHIP**  Ontario Health Insurance Plan | Record of services from health care providers that claim under OHIP | MOHLTC | - patient and physician identifiers (encrypted) - code for service provided, date of service, and associated diagnosis - fee paid | - IKN - Date of admission - OHIP fee code and suffix *or* diagnosis code and explanatory code | - Dates and diagnoses related to physician consultations for identification of study outcomes, and individual’s medical histories - January 1994-December 2009 | - 1 diagnosis per visit - 3-digit diagnosis code (variant of ICD-9) - 1-character ‘type of diagnosis’ code - 1-digit diagnosis code suffix |
| **IRIS**  Immunization Record Information System | Record of the immunization status of school-aged children and children in licensed child care facilities | Local Public Health Agencies | - student demographics (e.g. date of birth, sex, school) - vaccine type and immunization dates | - list of girls born in 1994 and 1995 for cohort - dates of HPV immunizations | - Identification of cohort through linkage to RPDB - Dates of HPV immunization for exposure status - January 1994-April 2010 | - N/A |

MOLTCH = Ministry of Health and Long-Term Care; ICES = Institute of Clinical Evaluative Sciences; IRIS = Immunization Record Information System; N/A = Not applicable; CIHI = Canadian Institutes for Health Information; ICD-9 = International Classification of Diseases, ninth revision; ICD-10 = International Classification of Diseases, tenth revision

# Appendix 2 Diagnoses and corresponding diagnostic codes for baseline medical history*

| **Diagnosis** | **ICD-9 code** | **ICD-10 code** | **OHIP code** |
| --- | --- | --- | --- |
| **Previous Diagnoses** | | | |
| Infectious and parasitic diseases | 001-139 | A00-B99 |  |
| Intestinal infections | 008-009 | A08-A09 |  |
| Chickenpox (varicella) | 052 | B01 | 025 |
| Neoplasms | 140-239 | C00-D48 |  |
| Metabolic disorders | 276 | E87 |  |
| Disorders involving the immune mechanism | 279 | D80-D89 |  |
| Diseases of the blood and blood-forming organs | 280-289 | D50-D89 |  |
| Mental disorders | 290-319 | F00-F99 |  |
| Epilepsy, recurrent seizures, convulsions | 345, 7803 | G40-G41, R56 | 345 |
| Non-infective gastroenteritis and colitis, unspecified | 558 | K52 |  |
| Nephritis, nephrotic syndrome, and nephrosis | 580-589 | N00-N05 |  |
| Congenital anomalies | 740-759 | Q00-Q99 |  |
| Syncope and collapse | 7802 | R55 |  |
| Other previous diagnoses |  |  |  |
| *Nutritional deficiencies* | 260-269 | E40-E46, E50-E64 |  |
| *Inflammatory diseases of the central nervous system (e.g., bacterial meningitis, encephalitis)* | 320-326 | G00-G09 |  |
| *Cerebral degenerations usually manifest in childhood* | 330 | G31-G32 |  |
| *Cerebral palsy and other paralytic syndromes* | 342-343 | G80-G81 |  |
| *Diseases of the circulatory system* | 390-459 | I00-I99 |  |
| *Pneumonia* | 480-486 | J12-J18 |  |
| **Immune-Mediated Events** | | | |
| Allergic rhinitis | 477 | J30 | 477 |
| Asthma | 493 | J45-J46 | 493 |
| Dermatitis and related conditions | 691-693 | L20-L30 |  |
| **Autoimmune Diseases** | | | |
| Diabetes mellitus | 250 | E11 | 250 |
| Multiple sclerosis (and other demyelination diseases of the central nervous system) | 340-341 | G35-G37 | 340 |
| Bell’s palsy | 3510 | G510 | 351 |
| Acute infective polyneuritis (including Guillain-Barré syndrome ) | 3570 | G610 |  |
| Rheumatoid arthritis and other inflammatory polyarthropathies | 7143 | M08 | 711, 714-716 |
| Systemic lupus erythmetosus | 7100 | M32 |  |
| **Risk Factors for Autoimmune Diseases** | | | |
| Cytomegalovirus | 0785 | B25 |  |
| Epstein-Barr virus | 075 | B270 |  |
| Campylobacter | 00843 | A045 |  |
| Influenza | 487-488 | J09-J11 | 147, 487 |
| Mycoplasma pneumoniae | 4830 | B960, J157, J200, P236 |  |

*Because physician visit codes often represent a number of different diagnoses, medical histories were based primarily on hospital and ED records.
